# Doing trials within trials: a qualitative study of stakeholder views on barriers and facilitators to the routine adoption of methodology research in clinical trials

**DOI:** 10.1186/s13063-018-2862-6

**Published:** 2018-09-10

**Authors:** Jo Rick, Mike Clarke, Alan A. Montgomery, Paul Brocklehurst, Rachel Evans, Peter Bower

**Affiliations:** 1 North West Hub for Trials Methodology, Manchester Academic Health Science Centre, University of Manchester, Williamson Building, Manchester, M13 9PL UK; 2 Northern Ireland Methodology Hub, Centre for Public Health, Queens University, Belfast, BT12 6BJ UK; 3 Nottingham Clinical Trials Unit Faculty of Medicine and Health Sciences, Queens Medical Centre, University of Nottingham, Nottingham, NG7 2UH UK; 4 NWORTH Clinical Trials Unit, Bangor University, Bangor, LL57 2PZ UK

**Keywords:** RCTs, SWATS, Methodology research

## Abstract

**Background:**

Randomised controlled trials are the cornerstone of evidence-based health care, yet many trials struggle with recruitment and retention. All too often the methodologies employed to address these problems are not evidence-based, as rigorous methodological research on these issues is rare. The current research sought to identify barriers to the routine implementation of methodology research around recruitment and retention.

**Methods:**

All registered UK clinical trials unit directors were sent a short questionnaire and invited to interview. Representatives of funding bodies and other stakeholders were also approached. Interviews were recorded and the content analysed.

**Results:**

Data were grouped into four themes: acceptance of the need for methodological research; trial funding and development; trial processes; and organisational factors. The need to improve the evidence base for trials methodology is well established, but numerous barriers to implementation were perceived.

**Conclusions:**

The knowledge and expertise required to routinely implement methodology research exists within the current research structures, and there are clear opportunities to develop the evidence base. However, for this to be achieved there is also a need for clear strategic coordination within the sector and promotion of the necessary resources.

## Background

Randomised controlled trials are fundamental to evidence-based practice. Increasing numbers of studies are being conducted worldwide, but recruitment and retention remain significant challenges. This highlights the importance of methodological work to improve trial delivery and quality.

One way of improving trials is through ‘research on research’: conducting methodology studies on the process of collecting and analysing data. ‘Studies within a trial’ (SWATs) embed research within research, so as to resolve uncertainties about the effects of different ways of designing, conducting, analysing and interpreting evaluations of health and social care [1]. They are potentially efficient and effective ways of rapidly developing the evidence base around the methodologies for trials, taking advantage of existing trial activity to conduct additional work. Such approaches are increasingly important in the context of concerns about research waste [2]. Other terms common in the literature include ‘embedded studies’, ‘nested’ studies or ‘methodological bolt-ons’. The term SWAT is used here to denote all methodology research embedded in trials.

The aspects of trial design and delivery tested in SWATs vary, ranging from modest, no-cost or low-cost comparisons, such as modifying patient information sheets [3], to more substantial endeavours (e.g. developing bespoke websites to support recruitment, training programmes for recruitment staff). Methods used in SWATs can include simple before-and-after studies and non-randomised comparisons, but our particular interest is in the subset of SWATs which themselves use a randomised design.

Despite the potential of SWATs to contribute to the evidence base on trial conduct, they are rare. For example, the Cochrane methodology review on recruitment interventions identified only 45 embedded methodology trials in real and hypothetical trials [4], while the partner review on retention reported only 38 trials, many of which focused solely on boosting the response to questionnaires [5]. Additionally, these reviews reveal the piecemeal nature of the evidence, with a variety of interventions tested in a range of trial contexts, with few replications.

Some of the ‘downstream’ barriers and facilitators amongst investigators to embed recruitment studies have already been explored [6]. A recent programme of work (Medical Research Council Systematic Techniques for Assisting Recruitment to Trials (MRC START)) evaluated two recruitment interventions by implementing 10 SWATs in existing host trials [7]. This work demonstrated the feasibility of the approach, but it also highlighted a number of barriers that had an impact on the decision to engage with SWATS. However, less is known about the wider structural and organisational factors that influence the implementation of SWATs.

Trials are developed and delivered over a long time scale, and our initial diagnosis of the barriers suggested that many could be addressed through early engagement in the trial development process, using ‘upstream’ interventions to overcome concerns on the part of investigators [6]. Important upstream influences are funders and clinical trials units (CTUs, academic organisations set up to specialise in the management and delivery of clinical trials). We require a comprehensive assessment of the organisational issues that might affect funders and CTUs and how they might relate to the concerns of those running trials.

The current study aimed to:

1. Identify potential barriers to and facilitators of the implementation of SWATs amongst trialists, funders and CTUs
2. Develop policies and guidance to support greater use of SWATs.

## Methods

### Sample and data collection

CTUs were established in the UK in 2007 as a way of bringing together staff with the core skills needed to deliver trials. CTUs focus on the design and delivery of trials, running trials in their own right and offering external teams a range of services from full trial management to support with design, data collection, data management, statistical services or information technology (IT).

Directors of all 44 CTUs registered as of June 2015 were sent a short screening questionnaire asking about SWAT activity and inviting them to an interview. Nineteen responded, and 12 (63%) interviews took place, involving 15 respondents (3 interviews involved 2 people). Key representatives of the main trial funders were also identified by the research team. Senior trialists and trial managers were identified through interviews with CTU leads or through their attendance at the UK Trial Manager Network (UKTMN) conference in September 2015. An additional 8 interviews were conducted with this second group (4 with individual trial managers, and 4 with funders, involving 5 respondents). In all, 24 people were involved in the interviews.

### Workshops and webinar

Two workshops were held during the course of the research: one at the UKTMN annual conference and one at the International Clinical Trials Methodology Conference (ICTMC), both in late 2015. A webinar, open to all UKTMN members, was held in December 2015.

### Data collection

We report the qualitative research in line with the Consolidated Criteria for Reporting Qualitative Research (COREQ) guidance [8]. All potential interviewees were sent a participant information sheet, consent form and covering email, asking them to participate in an interview. Interviews took place between July 2015 and January 2016. Interviews were predominantly conducted by phone by JR (female health services researcher with a PhD and experience with conducting interviews), using a semi-structured interview guide and were mostly digitally recorded and transcribed (a minority were recorded by hand). Interviews ranged in length from 30 to 90 min, with the majority lasting around an hour (average = 58 min). Most of the interviewees had no previous relationship with or knowledge of the researcher prior to the study. Additional material was collected via workshops at two conferences with trial managers, academics and clinicians with an interest in methodology, and via a webinar hosted by the UKTMN. Notes were taken at the workshops and webinar and used to inform interpretation of results.

### Data analysis

Interviews were transcribed and entered into NVivo. Coding was done by one researcher (JR). We did not return transcripts to participants for comment, nor did we ask them to comment on the findings. As noted earlier, our preliminary work suggested that implementation of SWATs amongst trialists would be affected by the operation of two key organisations: funding bodies and CTUs. Transcripts were thus coded using a framework of relevance to understanding organisational function. The McKinsey 7S model was originally developed as a model of organisational change [9]. It is a useful framework for understanding how organisations deal with multiple priorities and pressures. McKinsey 7S identifies seven interrelated factors or elements that influence an organisation’s ability or readiness to implement a strategy (strategy, structure, systems, shared values, staff, style and skills). The model is relatively simple and suitable for use in varying contexts. It is used here as a framework through which to consider the organisational aspects of CTUs that can act as barriers or facilitators to SWATs.

## Results

Data were grouped into four broad themes:

1. Understanding and acceptance of the need for SWATs
2. Wider context of trial development and funding
3. Specific stages of trial development, implementation and delivery
4. Organisational aspects of CTUs (using the McKinsey 7S model)

### Understanding and acceptance of the need for SWATs

Recognition of the need for SWATs was well established amongst CTU leads, senior trialists and trial managers interviewed here. As one senior trialist commented:

*… for at least 50 years people have struggled with the idea of making trials as efficient as they can be…and every time you looked at the literature, people were still struggling … and they still are…clearly things are not improving in the way we think they should.*

However, this recognition was not seen to extend to the wider trial community, where Chief Investigators (CIs) or other trial management group members were frequently cited as barriers to employing SWAT methodology. The types of concerns encountered tended to focus on resisting further complexity, increasing workload, a belief in a particular methodological approach (lack of equipoise) or fear of reducing recruitment in one arm:

*I did raise it with one CI … around payment for follow up data … they said ’we don’t want a randomised payment or no payment because we believe it works’ … they wanted to give it to everybody because they don’t want to risk reducing the recruitment in one arm…*

*I did put in a section [on the application] saying we are going to do an embedded trial, but all the other applicants thought this was a distraction and wanted me to take it out, which I did*

Good relationships (normally between CTU and CI and occasionally between trial manager and CI) and shared values around methodology research were cited numerous times as important facilitators:

*If you’ve got a long standing relationship, that’s fine, but if they are a new CI you might not want to raise it necessarily.*

Opinion was divided on the role of academic clinicians in promoting or supporting embedded methodology research and SWATs:

*I think clinician CIs, because they are less experienced can go along with whatever you say, whereas academics, who maybe feel they could get the grant in their own right, are more likely to want to do what they want…*

Another potential facilitator of SWATs was the extent to which CTUs could make inclusion of a SWAT conditional for accepting a trial or application, and the continued promotion of this type of work through dissemination, networking and support.

Some interviewees questioned the need for or suitability of SWATs. Exceptions included examples of improved trial procedure that they did not feel needed to be tested:

*Within the CTU we’re always looking at how we can improve our systems and processes of running trials, we just don’t do it by, sort of, formally randomising to do method A or method B … We know it’s working because we know it happens quicker…*

Some trial settings were seen as inappropriate for a SWAT:

*A lot of the time we’re recruiting patients the night before they go in for major surgery … so it’s difficult to imagine what we could embed methodologically given the sort of patient pathway we work with most of the time.*

Finally, there were mixed comments from funder representatives, with one contending that SWATs are too narrow in focus: the approach misses key determinants of successful trials. Issues are not how to recruit, but management style and leadership:

*My impression of the literature … is that it’s adopted a rather too narrow study design paradigm … in terms of fixed interventions and …randomised controlled trials. I think there’s often modest differences between individual interventions and the trials that are successful are not because they’ve adopted a particular approach … it’s because they’ve been just really well organised*

Other funder views aligned more with those of CTU leads, acknowledging the need to improve the evidence base on recruitment and retention strategies.

### Wider trial development and funding context

Substantial differences existed in perceptions about availability of funding for methodology work. For those engaged in delivering research, difficulty in accessing funds was the most significant barrier. None of the current funding streams were seen as open to funding stand-alone SWAT applications:

*So we think it’s a good thing, and also one of our other objectives is to do more methodological work and there aren’t many funding streams for methodological work. In fact I can’t really find any, so…*

Views from representatives of funding agencies were mixed, but on the whole reflected a more positive view, citing examples where the opportunity for ‘methodological bolt-ons’ existed:

*The [methodological bolt-ons] were put on there in the sense that if people are funded to do a trial using some new method … is there some additional research that might be done to either evaluate or test the validity of the approach that is being done? So, we haven’t any more thoughts on that …*

Some funders reported shifts in policy towards more applied work:

*It was decided to look at whether there should be changes to the structure and membership of the [Panel] to try and make sure we got more of an applied bent … it was an attempt to increase [applied methodology research] but of course we’re entirely reliant on the applications that come to us*

Outside these programmes, there was some indication from funders that applications for SWATS as part of a full trial funding application could be considered ‘in scope’:

*But, you know, something of modest scale that represents an internal evaluation of recruitment methods, in order to inform the conduct of the later phase of the trial, seems entirely reasonable.*

However, from the perspective of trialists, there was not always clarity about access to funds:

*I guess knowing that the funder actually wants to see it and is prepared to pay for it and upfront explicitly say, because otherwise you’ll just think, oh well they’ll say, why have you put this extra thing in, it just makes it more complicated and more expensive?*

From a funder perspective that meant clearer communication about funding for SWATs:

*Interviewer: There seems to be a perception that it’s [SWATs] not something [funder] would fund …*

*Respondent: Yes … I think it’s an incorrect perception, but a widely held perception*

Several other contextual factors were identified by researchers as barriers to the adoption of SWATs. Timing pressures in the trial funding cycle were commonly mentioned as the main factor working against the added complexity of developing and introducing SWATS:

*At the moment the way they’ve got the expression of interest to the full in seven weeks, either over Christmas, over summer or over Easter is just not giving you the opportunity to start being creative about method study.*

However, several interviewees noted that efforts to co-ordinate ideas and develop ‘off-the-shelf’ interventions could act as a facilitator in this setting.

A second barrier of considerable concern relates to costs and competition. Many perceive current funding strategy as placing great emphasis on cost saving, value for money and efficiency. Ironically, in this context CIs and CTUs tend to strip design back and are keen to ensure no extraneous costs in funding proposals, acting as a barrier to SWATs. CTU leads in particular expressed concerns about whether additional costs for SWATs could have an impact on their competitiveness:

*I think like with any trials unit we are always being pushed back on our costs, and our costs, I would argue, are actually very competitive. There’s always that pressure to do all that you want to do for less because of the fear that it will get knocked back by the funding board if it’s seen to be too expensive.*

### Specific stages of trial development, implementation and delivery

There were many comments about the detailed process of implementing a SWAT. A number of key stages of trial development, implementation and delivery were noted, but a considerable proportion related to activities that could be considered as earlier stages. Moving from support for the principle to idea generation for a specific SWAT was one area of difficulty. Concerns related partly to timing (as noted above) but also the broader question of what constitutes a worthwhile intervention to test:

*Yes. And I think the other element to all of that is the timing of it all. We’ve had a lot of discussions about how much of a time window we have between people approaching us and then a funding deadline, so it’s then on top of all of what you have to do anyway, building in this. It’s whether you’ve got something ready to go and you think, oh, this trial would be perfect, or whether it’s developing something from scratch.*

There was particular support for some form of agenda-setting for research priorities and co-ordinating efforts around specific questions to have maximum impact, possibly forming collaborations with small groups of CTUs:

*Yes, one of the key things I always talk about with this Trial Forge idea which is sort of distilling down now to essentially one line, three words, it’s ‘coordination and collaboration’. The first bit is that coordination, it’s what should we do? Again that removes a barrier if you can say, well, here are some things to do, and in fact if we take the IQuaD example, not only: here are some things, but here is the thing to do. It removes a barrier, you just make a decision, do I want to take part or not? You haven’t had to dream the thing up.*

Host trial characteristics, stage of trial development and the level of service provided by the CTU were seen as critical for determining suitability for a SWAT. The complexities associated with running a SWAT were felt to be reduced where CTUs provide a full trial service (as opposed to data management only) and where they are involved in the design and development of the trial from an early stage. Other potential facilitators included checklists or guidance for identifying suitable host trials, detailing what needs to be taken into account when negotiating access for the SWAT:

*In X CTU you buy the whole service. They are very, very reluctant to take on a trial if they are not running it completely, so there is the potential to bolt stuff on. …in Y CTU there is a much wider range of support …[they] would run things completely but also there were investigators who would just buy data management … so if they’re doing trial management themselves and you suggest doing an embedded methodological study that will affect the working life of their trial manager, I think you’re going to struggle …*

External review as part of funding or ethics applications was seen as problematic, with a SWAT seen as having the potential to cause confusion, detract from the main trial or, at worst, generate objections that undermine the host trial or delay its implementation. Providing directions from funders to peer reviewers on how SWATs should be assessed was seen as useful:

*Funders and the referee, it’s not just funders, it’s referees get…and they might, sort of, focus on making hugely critical comments on an embedded trial that they profoundly disagree with or something and that might damage the whole…there’s a fear it might damage the whole proposal.*

Greater uncertainty was expressed about how SWATs would be reviewed within ethics, with a general sense that a SWAT could cause complications. This was felt particularly where SWAT methodology might diverge from standard practice. Examples included cases where there is no pre-notification of participants in a SWAT or where a power calculation for a SWAT is less appropriate.

Potential facilitators included engaging with the ethics committees to discuss whether guidance could be given on how this type of work should be treated:

*Some [research ethics committees ] still specialise in drugs, some will specialise in radiotherapy, some around consent for children…why not have a few ethics committees think about embedded methodology, or methodology in general?*

The major barriers perceived by researchers in relation to trial process and conduct were focused on the early phases of the research process (concept, development, funding and review). However, a number of concerns relating to trial implementation were also identified. Trial setup is a time-critical period in the life of the trial. Reservations were expressed about the practicality of running a SWAT if it added to the burden of setup. Various solutions were suggested, including delaying the start of the SWAT or using a design (such as cluster randomisation) that simplified the recruitment work on site, although there were concerns that these types of actions could in themselves cause complications:

*…it’s to do with the centre of gravity, they don’t want the centre of gravity of the project to shift from what they are actually really interested in. So if … you have a substantial chunk of your money and effort is refocused on a methodological piece of work, you are starting to move the centre of gravity away from what they [funders/main trial] are really interested in and that won’t fly.*

There was also concern about monitoring and implementation fidelity of the embedded trial, particularly on large multi-site trials where the CTU or trial team might be remote from day-to-day recruitment practice. It was felt that this would have to be considered when selecting host trials.

A final concern raised was over ownership of the embedded trial research and data generated and the need to establish this early in the process.

### Organisational aspects of CTUs

CTU lead interviewees were asked to consider barriers and facilitators to embedded methodology research within their own organisations and networks. Responses were analysed using the McKinsey 7S model and related to five aspects: strategy, skills, structure, style and systems.

#### Strategy: plan or course of action leading to the allocation of an organisation’s finite resources to reach identified goals

The potential of SWATs to contribute to CTU strategy was identified in a number of key areas such as increasing unit capacity, staff skills and methodology work. However, the extent to which SWATs formed part of CTU strategy varied considerably. Examples ranged from explicit and enacted strategy in one CTU to different stages of consideration or implementation:

*Well it’s across the board, we try and do it on every trial…*

*Okay, well we’ve been thinking about it for a long time because we’re obviously aware that other people do it, and some people get a lot of papers out of it, and also it’s a potential for trial managers or trial staff to get their own papers to lead on a paper and be a first author. So we think it’s a good thing, and also one of our other objectives is to do more methodological work*

This included in some cases testing the idea with external collaborators or as part of a broad strategy to expand, increase skills and capacity, and forge links with other university departments:

*So as I said with this…this was the first kind of formal raising of it [SWATs] at this meeting [with a group of collaborators] and basically it got very positive feedback and I think it will get positive feedback from the others [collaborator group meetings] as well, and I think part of the reason for that is that we have… but if you look on our website you will see that we are going to build up a very strong track record of methodology*

There was also widespread recognition of how the CTU network could be used to facilitate greater strategic commitment to SWATs:

*I mean, perhaps it should be…we should try and persuade one or two others just to start doing this [SWATs] and then perhaps it would then, you know, percolate around more.*

However, given the operating pressures within CTUs, it was unclear whether such additional work would be undertaken without further strategic drivers, primarily around access to funding and perceived importance. There was awareness of new initiatives such as Trial Forge [10] and the SWAT repository (go.qub.ac.uk/SWAT-SWAR); however, few reported engagement, and the links between these aspects of the trials infrastructure are only just being made.

#### Skills: distinctive capabilities of key personnel and the organisation as a whole

As might be expected, skill profiles across CTUs are broadly similar, with core staff including statisticians, methodologists and data managers. Running SWATs was felt to be well within the existing CTU skill set, both in terms of individual and organisational skills:

*We have all the team we need really, trial managers, data managers, the usual randomisation services, databases, statisticians. Then we liaise with health economists*

Two aspects of CTU staffing were seen as intrinsic to successful SWAT implementation and delivery, both focused on the role of trial managers. Developing trial manager skills was largely described in terms of on-the-job training, with SWATs recognised as offering important opportunities for staff to experience all aspects of trial management and improve career options both through skill development and lead authorship on SWAT publications:

*My main driving force is to help my trial managers to get publication for their CVs, that’s one of the main reasons for doing embedded trials, as well as it’s nice to generate the evidence, but it’s also nice to get them through promotions committees which are easier to do if they’ve got publications on their CVs.*

The second set of comments in relation to staff concerned the location of trial managers. There are much better prospects for implementing SWATs where trial managers are based in a CTU or closely linked department, and several CTUs insist on this as a condition of taking on a study:

*…it will always be really important for us to have an academic co-applicant on external studies so that we are in a position as a CTU to be able to influence the overall shape and design of the study…*

CTUs operating a more remote model (e.g. with no co-investigator on the trial or where they provide a single service, such as data management) saw this as a considerable barrier.

#### Structure: who is in charge, how decisions are made, authority relationships

CTU leads all described broadly similar decision-making structures within their organisations, with a board or senior management team assessing each clinical trial proposal. However, for SWATs, decision-making power does not necessarily sit with the CTU. This can affect their ability to make direct decisions about implementing SWATs in a particular trial where the CTU is dependent on a receptive CI:

*One of the problems when you’re just doing it purely service, is this issue around how much control you have, and CIs are very different. Some are very happy to have all the input of a CTU and in some ways actually allow the CTU to effectively lead the study. Others it’s the opposite*

It can also have an impact on broader decisions about CTU strategy:

*I know some other units … have their own cost centre and are, like, independent organisations in the universities, but we’re not. So we’re in a bigger department and that has its up and downsides, I mean, the upside is that we have access to other specialties, other people more easily and we can spread the risk sometimes of if we have a short fall in work, sometimes people can be moved to projects within the department. I suppose the downside is we have less control of our own destiny.*

#### Style: characterisation of how key managers behave in order to achieve the organisation’s goals

There was strong recognition amongst interviewees of the importance of senior management support if SWATs are to be more widely adopted. This ranged from creating a culture of expectation that all trials will seek to adopt SWATs, to seeking and promoting specific opportunities to work collaboratively:

*If you look at XXXX [CTU]… the director of that unit will support trial managers who are interested in running a piece of [methodological] research to do so and to get a publication out of it… my take on it is that a) it’s supporting the methodological literature, but b) it’s also supporting the individual trial manager and it’s part of the culture. If you’re in a different unit where [the leadership] see the trial manager’s role as someone who essentially implements the trial, it might be much harder.*

#### Systems: procedures and routine processes, including how information moves around the organisation

The nature of trials work means that CTUs have high levels of expertise in developing and implementing systems and offer great scope for routinizing SWATs. However, the lack of SWATs means that, in practice, these systems rarely exist. Additionally, because most SWATs have been conducted in an ad hoc way to date, there is little shared learning on what processes seem to work best.

Looking at existing systems and identifying where SWAT elements could be included was felt to be a straightforward process for CTUs, but it was also felt to be a minor consideration in relation to the other barriers faced, such as funding, consensus around what to test and resources to develop ideas and materials.

## Discussion

Awareness of recruitment and retention issues and acceptance of the need for SWATs amongst the wider research community remains a considerable barrier to the wider adoption of SWATs. There is also a gap between the perceptions of funders and researchers about the availability of funding. A number of simple actions could remedy this: greater clarity from funders about the status of funding for SWATs; actions that send clear signals to trial teams and beyond about support for SWATs (changes to application forms to outline any SWATs included in the trial); and greater willingness from CIs and methodologists to discuss proposals for SWATs with funders prior to submission of a bid.

### Limitations

It is recognised that SWAT methodology will be more or less relevant for certain types of trials and settings. Whilst all interviewees here broadly supported the SWAT approach, two interviewees also highlighted the importance of other approaches to improving trial methodology, including improving internal CTU systems or focusing on leadership style and management of trial teams [11, 12], that may not require testing through embedded methodology research.

The sample in this research is small and to some degree self-selected. In particular, the representatives of trial funders may not reflect the views or priorities of the funding organisations as a whole. The views expressed here relate to the present delivery of publicly funded trials in the UK and may suffer from limited external validity to other contexts where funding and CTU arrangements may differ. Note also that priorities for methodological research differ between settings, with recruitment and retention less of an issue in low- and middle-income countries compared to outcome assessments [13].

### Implications

Real concerns exist about the balance between SWATs and host trials. Existing arguments have tended to focus on the lack of trial methodology evidence per se and the rationalisation therefore that SWATs are ‘a good thing’ in general. Implementation of SWATs will be aided by the development of more sophisticated arguments that demonstrate better awareness of the balance between the needs of a SWAT and the host trial and make explicit the synergy between a SWAT and host trial, including known or anticipated recruitment or retention challenges in the host population and use of designs, such as an adaptive embedded trial design, with clear indication of how and when the host trial team will review recruitment data and switch to the preferred recruitment method identified by the SWAT.

Although trialists and CTUs are well equipped (in terms of skills) to implement SWATs, the processes are often poorly understood. Additionally, there are areas where SWAT methodology diverges from standard trial practice. Various materials exist via current initiatives, such as the SWATs repository and Trial Forge [10, 14], that could form the basis for a set of guidelines for running SWATs.

The CTU network offers unique opportunities to develop a clearer strategy with regard to embedded methodology trials. To date, efforts have been ‘piecemeal’ and opportunistic, but there is recognition that CTUs could develop a clear strategy which could be achieved in a number of ways: consensus about intervention priorities; collaboration between a number of CTUs to promote and implement interventions with a specific evidence goal in mind; or efforts from an individual CTU to promote and implement an intervention over a number of trials with regard to a specific recruitment process or question.

## Conclusions

The need for evidenced-based approaches to trial delivery is well established amongst the interviewees in this study. The CTU network already has the skills and expertise necessary to implement the widespread adoption of evidence-based approaches to trial methodology. However the approach, whilst appealing on a number of levels, is not without challenges. If SWAT methodology is to move beyond a fringe activity, there needs to be considerable coordination of effort across a number of linked activities: promoting wider understanding of the need for evidence-based trial methodology and setting expectations, particularly through funding policy, ethical review guidance and other governance standards; encouraging formal and informal collaboration on a much wider scale to agree on priorities for testing and setting agendas for development of evidence in specific issues; and formal and informal sharing of expertise and materials to support the implementation of SWATs.

## Abbreviations

CI: Chief Investigator
CTU: Clinical trials unit
ICTMC: International Clinical Trials Methodology Conference
SWAT: Study within a trial
UKTMN: UK Trial Manager Network
